# Validation of Selected Non-Destructive Methods for Determining the Compressive Strength of Masonry Units Made of Autoclaved Aerated Concrete

**DOI:** 10.3390/ma12030389

**Published:** 2019-01-26

**Authors:** Radosław Jasiński, Łukasz Drobiec, Wojciech Mazur

**Affiliations:** Department of Building Structures, Silesian University of Technology; ul. Akademicka 5, 44-100 Gliwice, Poland; lukasz.drobiec@polsl.pl (Ł.D.); wojciech.mazur@polsl.pl (W.M.)

**Keywords:** autoclaved aerated concrete (AAC), compressive strength, shape and size of specimen, moisture of AAC, ultrasonic testing

## Abstract

Minor-destructive (MDT) and non-destructive (NDT) techniques are not commonly used for masonry as they are complex and difficult to perform. This paper describes validation of the following methods: semi-non-destructive, non-destructive, and ultrasonic technique for autoclaved aerated concrete (AAC). The subject of this study covers the compressive strength of AAC test elements with declared various density classes of: 400, 500, 600, and 700 (kg/m^3^), at various moisture levels. Empirical data including the shape and size of specimens, were established from tests on 494 cylindrical and cuboid specimens, and standard cube specimens 100 mm × 100 mm × 100 mm using the general relationship for ordinary concrete (Neville’s curve). The effect of moisture on AAC was taken into account while determining the strength *f*_Bw_ for 127 standard specimens tested at different levels of water content (*w* = 100%, 67%, 33%, 23%, and 10%). Defined empirical relations were suitable to correct the compressive strength of dry specimens. For 91 specimens 100 mm × 100 mm × 100 mm, the P-wave velocity *c*_p_ was tested with the transmission method using the ultrasonic pulse velocity method with exponential transducers. The curve (*f*_Bw_–*c*_p_) for determining the compressive strength of AAC elements with any moisture level (*f*_Bw_) was established. The developed methods turned out to be statistically significant and can be successfully applied during in-situ tests. Semi-non-destructive testing can be used independently, whereas the non-destructive technique can be only applied when the developed curve *f*_bw_–*c*_p_ is scaled.

## 1. Introduction

Significant variations in materials, technology, and performance cause that masonry structures are much more difficult to be diagnosed than concrete or reinforced concrete, for which the standard EN 13791:2011 [1] specifies both the methodology of tests and conclusions. Regarding masonry structures, there are no standards that classify testing methods. Methods which directly determine compressive or shear strength of a wall, are commonly assumed as destructive testing (DT). Those studies consist in testing fragments of a masonry wall [2,3]. Destructive (direct) techniques use fragments of walls or flat jacks in bed joints, and deliver test results in the form of compressive strength of the wall *f*_k_. These methods cause quite a significant damage to the wall. Consequently, the number of tests to be performed becomes sharply limited.

Non-destructive testing (NDT) conducted on masonry walls, which is per analogiam to reinforced concrete structure, include the following methods: sclerometric method, ultrasound method, and pull-out method, which are not commonly used and have not been normalized so far [4,5,6,7]. There are some recommendations [8] and general guidelines, but they are not regarded as the European document. Tests can be performed to evaluate compressive strength providing that the appropriate standard curve will be scaled taking into account destructive tests conducted on cores from the structure (or on masonry units, or the mortar). The number of tests conducted with these techniques is significantly high, the damage of masonry structures is not severe and can be easily repaired. Unfortunately, there are no standard curves for adequate scaling except for original solutions.

Minor-destructive testing (MDT) is most commonly applied for masonry structures. This technique consists of taking small cores from such a structure or applying flat-jack. As it is in the case of NDT, there are no uniform regulations at the European level. The practical application of flat-jack technique involves American standards [9,10,11,12]. For small cores from the wall structure, some recommendations [13], which specify conversion factors for solid brick wall to determine its compressive strength. Besides, compressive strength can be determined on the basis of tests conducted on wall components (masonry units and the mortar). This technique consists of converting compressive strength of small specimens into the strength of standard specimens (*f*_b_ into *f*_m_), and using standard equations in their exponential form *f*_k_ = *Kf*_b_^α^*f*_m_^β^ (*K*—coefficient specified in EC-6). There are not many tests in this field, and the performed ones are rather single cases [14,15,16] and usually refer to solid brick and traditional mortar. Non-destructive and semi-non-destructive tests are indirect techniques because they do not determine compressive strength of the wall, but the strength of its component (masonry unit or mortar). Neither NDT nor MDT techniques can be used to determine compressive strength of the wall without performing destructive tests to scale the suitable correlation curve to convert obtained strength values into the requested value *f*_k_ [17].

Determining the compressive strength of modern masonry walls with thin joints, where mortar levels any irregularities of support areas and head joints are unfilled, requires only the properly determined compressive strength of the masonry unit *f*_B_ and calculated (with empirical factors *η*_w_ and *δ* expressing the specimen moisture and shape) an average normalized compressive strength. This procedure involves the relationship according to Eurocode 6 [18], and is used to calculate the specific compressive strength of the masonry wall:

(1) $$f_{k}=Kf_{b}^{0.85}=K{\left(\eta_{w}\delta f_{B}\right)}^{0.85}\to K{\left(f_{\mathrm{Bw}}\right)}^{0.85}$$

where *K* = 0.75 or 0.8, *f*_b_—average normalized compressive strength of masonry unit determined for specimens 100 mm × 100 mm × 100 mm, *f*_B_—average compressive strength of the whole masonry unit or a specimen with moisture content *w* = 0, *f*_Bw_—compressive strength of the specimen from the masonry with real moisture content.

If tests are performed on specimens having different dimensions than a cube with a 10 mm side, the normalized strength is determined using *δ* factors specified in the standard PN-EN 772-1 [19]. However, the standard does not specify conversion factors for non-standard specimens, such as cores or micro-cores. Consequently, the conversion of results is burdened with a default error that is difficult to be estimated. The literature [20,21,22] describes conversion factors obtained from tests on other materials, such as concrete, ceramics, or masonry units [23]. No relations to AAC have been presented so far. 

Autoclaved aerated concrete (AAC) contains cement, calcium, and lime as binding material, sand used as a filler and tiny quantities of aluminium powder (or paste), which is used as a blowing agent. Density of this type of concrete ranges from 300 to 1000 kg/m^3^, and its compressive strength varies from 1.5 to 10 N/mm^2^. Taking into account all construction materials, AAC is characterized by the highest thermal insulation power (thermal conductivity coefficient λ is 8–10 times lower compared to brick or reinforced concrete). AAC has been commonly used since the middle of the 1950s. This material (>40% of the construction segment in Europe) is used for masonry structures, precast wall or floor elements, and lintels [24]. The open-pore structure explains why AAC is sensitive to direct exposure to moisture, which results in worse insulating and strength properties. The available articles, apart from general relations specified in standards, do not contain detailed references expressed as empirical relations.

There are no procedures for determining specific compressive strength of the existing masonry wall with the actual density and moisture content. However, in some situations, drilling micro-cores, and even performing sclerometic tests is impossible. Therefore, only ultrasonic non-destructive techniques can be used to determine compressive strength.

The main aim of this article was to present the complex analysis of strength issues, which included developing empirical relations to determine compressive strength *f*_b_ on the basis of tests performed on specimens of any shape and real moisture content, and to develop the universal curve representing ultrasound velocity *c*_p_ and compressive strength, taking into account moisture content.

This article describes an attempt to establish the empirical curve for determining the normalized compressive strength of the AAC masonry unit, with unspecified density and moisture content *f*_Bw_ using semi-non-destructive techniques. Neville’s curve [20], in the commonly known form from diagnosing ordinary concrete, was used and calibrated to nominal density classes of AAC (400, 500, 600, and 700). Knowing that, apart from the effect of rising and hardening [25,26], also moisture content in AAC influences the compressive strength, tests were performed and additional empirical relations were defined. The analysis included test results [27] from 494 + 127 cylindrical and cuboid specimens used to develop empirical curves. Results obtained from destructive tests on standard cube specimens 100 mm × 100 mm × 100 mm at different moisture content were correlated with results from testing velocity of P-wave generated by point transducers with the transmission method. Developed curves and the test procedure can be employed in a widely understood diagnostic of masonry structures to evaluate the safety of AAC structures.

## 2. Minor-Destructive Testing

### 2.1. Specimens, Technique of Tests, and Analysis

**Figure 1:**
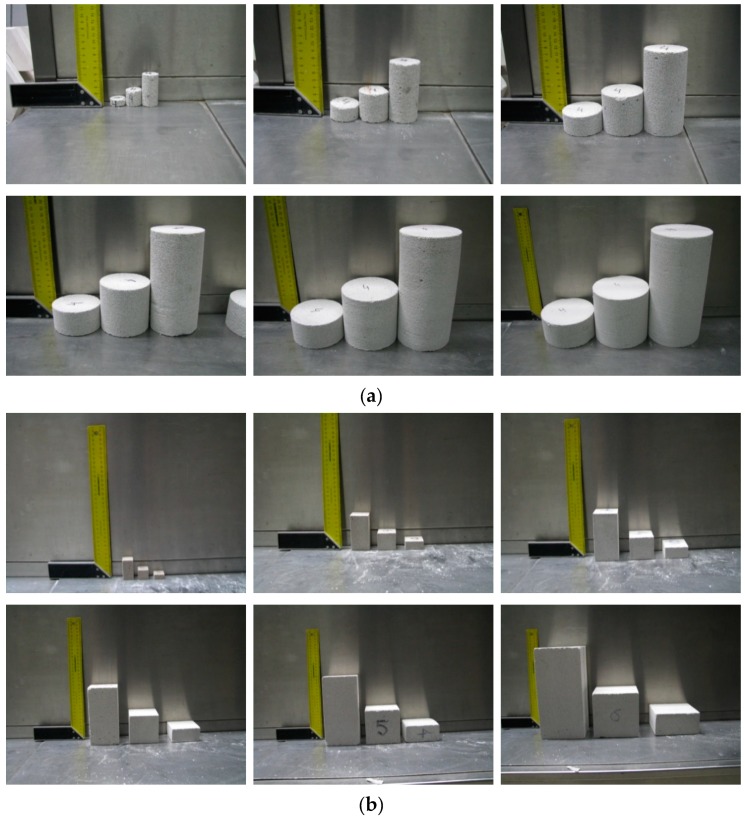
Specimens before tests [27]: (**a**) core specimens, and (**b**) cube specimens.

Tests included four series of masonry units with thickness within the range of 180–240 mm and different classes of density: 400, 500, 600, and 700, from each 20 masonry units were randomly selected. Six series of cores with varying diameters were taken from each masonry unit. Six series of square specimens having different side length and height were drilled from masonry units using a diamond saw. Cuboid specimens included blocks with dimensions of 100 mm × 100 mm × 100 mm, which were used as basic specimens for determining the strength *f*_B_ (in accordance with Appendix B to the standard EN 771-4 [16]). Drilled core and cube specimens are illustrated in Figure 1. All specimens drilled from blocks were dried until constant weight at a temperature of 105 °C ± 5 °C (for at least 36 h).

**Figure 2:**
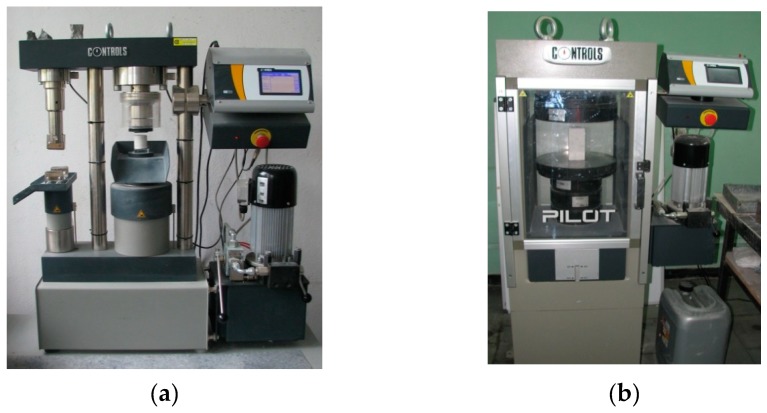
Testing compressive strength of AAC specimens [27]: (**a**) tests on cores using a strength testing machine with an operating range of 100 kN, and (**b**) tests on cuboid specimens using a strength testing machine with an operating range of 3000 kN.

Depending on the specimen size, loading rate was 2400 N/s or 100 N/s. Due to the size of specimens, two types of machines having an operating range of 100 kN and 3000 kN, and the class of accuracy of 0.5, were used (according to EN 772-1 [28]; Figure 2). Compressive strength *f*_B_ was determined for cube specimens 100 mm × 100 mm × 100 mm (dried until constant weight). The summary of our test results for core and cube specimens is shown in Table 1 and Table 2. Tables show dimensions and strength of each tested specimen, average strength and coefficient of variation for each tested series. Arrows indicate the direction of AAC growth. When dried until constant weight, each cuboid specimen was weighed and its apparent density was calculated (Table 3). 

**Figure 3:**
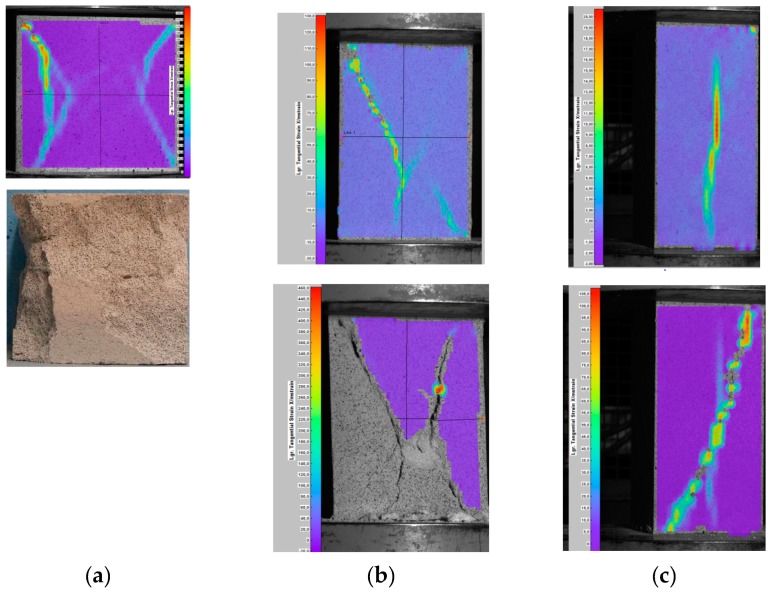
Destruction of specimens with varying slenderness ratio [27]: (**a**) specimen 143 mm × 143 mm × 143 mm, (**b**) specimen 100 mm × 100 mm × 200 mm, and (**c**) specimen 80 mm × 80 mm × 158 mm.

Development of cracks in cuboid specimens of different dimensions was recorded with an optical measuring system (Figure 3). In dense specimens with slenderness ratio *h*/*b* = 1, diagonal cracks developed at the upper edges, and they formed two truncated pyramids at failure (Figure 3a). In specimens with slenderness ratio *h*/*d* = 2, a vertical crack in the mid-length of the base appeared first, and then secondary diagonal cracks formed near corners of specimens (Figure 3b). The arrangement of cracks in specimens of bigger volume at failure was similar to dense specimens (Figure 3c). 

### 2.2. Determining an Empirical Curve in Air-Dry Conditions

If strength of the material depends on its defects, such as pores or voids, then individual specimens of different shapes can have significantly different values. These aspects are covered by Weibull’s statistical theory of material strength [29,30], which states that strength of the material is reversely proportional to the volume of the tested specimen at the same probability of failure:

(2) $$\frac{\sigma_{1}}{\sigma_{2}}={\left(\frac{V_{2}}{V_{1}}\right)}^{1/m}$$

where *σ*_1_ and *σ*_2_ are failure stresses for specimens with volume *V*_1_ and *V*_2_, respectively; m is constant.

The exponential type of Equation (2) is similar to hyperbole and is used during tests on compressive and tensile strength of dense specimens. Neville [20] developed a similar hyperbolic relation with regard to its course, while testing specimens of different slenderness. This relation is used to determine compressive strength of concrete in specimens with shape and dimensions different from those of standard specimens (blocks 150 mm × 150 mm × 150 mm). The empirical curve for ordinary concrete is expressed as:

(3) $$\frac{f_{c}}{f_{\mathrm{c,cube}\;150}}=0.56+\frac{0.697}{\frac{V}{152hd}+\frac{h}{d}}$$

where *V* is specimen volume, *h* is specimen height, and *d* is the smallest side dimension of the specimen.

Replacing strength *f*_c,cube150_ obtained from standard specimens 150 mm × 150 mm × 150 mm with strength *f*_B_ for specimens 100 mm × 100 mm × 100 mm drilled from masonry units, and the ratio 152*hd* with volume of the standard specimen 100*hd*, the relationship (3) can be expressed as:

(4) $$\frac{f_{c}}{f_{B}}=b+\frac{a}{\frac{V}{100hd}+\frac{h}{d}}\to y=b+\frac{a}{x}$$

where *f*_B_ is the compressive strength of normalised specimen 100 mm × 100 mm × 100 mm with moisture content *w* = 0, *f*_c_ is the compressive strength of a specimen with any shape and dimensions, and moisture content *w* = 0, *a* and *b* are constant coefficients for the curve, $y=f_{c}/f_{B}$ is the ratio of compressive strength, and x=V/(100hd)+h/d is the dimensionless coefficient representing the effect of specimen volume and slenderness. 

Requested parameters of the curve (4) were determined by searching a local minimum sum of squares difference:

(5) $$S\left(a,b\right)=\sum_{i=1}^{n}{\left[y_{i}-y\left(x_{i}\right)\right]}^{2}=\sum_{i=1}^{n}{\left[y_{i}-\left(\frac{a}{x_{i}}+b\right)\right]}^{2},$$

using the following relationships:

(6) $$\frac{\partial S\left(a,b\right)}{\partial a}=0,$$

(7) $$\frac{\partial S\left(a,b\right)}{\partial b}=0.$$

When the system of linear equations was differentiated and solved, the following relations were obtained expressed in the form facilitating the construction of a correlation table:

(8) $$a=\frac{\sum_{i=1}^{n}\frac{y_{i}}{x_{i}}-\frac{1}{n}\sum_{i=1}^{n}y_{i}\sum_{i=1}^{n}\frac{1}{x_{i}}}{\left(\sum_{i=1}^{n}\frac{1}{x_{i}^{2}}-\frac{1}{n}\sum_{i=1}^{n}\frac{1}{x_{i}}\sum_{i=1}^{n}\frac{1}{x_{i}}\right)},$$

(9) $$b=\frac{1}{n}\sum_{i=1}^{n}y_{i}-\frac{1}{n}\left(\frac{\sum_{i=1}^{n}\frac{y_{i}}{x_{i}}-\frac{1}{n}\sum_{i=1}^{n}y_{i}\sum_{i=1}^{n}\frac{1}{x_{i}}}{\left(\sum_{i=1}^{n}\frac{1}{x_{i}^{2}}-\frac{1}{n}\sum_{i=1}^{n}\frac{1}{x_{i}}\sum_{i=1}^{n}\frac{1}{x_{i}}\right)}\right)\sum_{i=1}^{n}\frac{1}{x_{i}}.$$

For defining compliance of the curve, some uncertainty was assumed to be neglected during measurements *x* (the specimen geometry). Additionally, uncertainties of all *y* values were the same (the same significance of measurements resulting from identical measuring techniques). To estimate the coefficient of correlation, the following values were calculated:

-error of estimate

(10) $$S_{\mathrm{tN}}=\frac{1}{n}\sum_{i=1}^{n}{\left(y_{i}-y_{m}\right)}^{2},$$

where: $y_{m}=\frac{1}{n}\sum_{i=1}^{n}y_{i}$,

-sum of errors:

(11) $$S_{\mathrm{rN}}=\frac{1}{n}\sum_{i=1}^{n}{\left(y_{i}-\left(\frac{a}{x_{i}}+b\right)\right)}^{2},$$

and then coefficient of correlation:

(12) $$R=\sqrt{\frac{S_{\mathrm{tN}}-S_{\mathrm{rN}}}{S_{\mathrm{tN}}}}.$$

**Figure 4:**
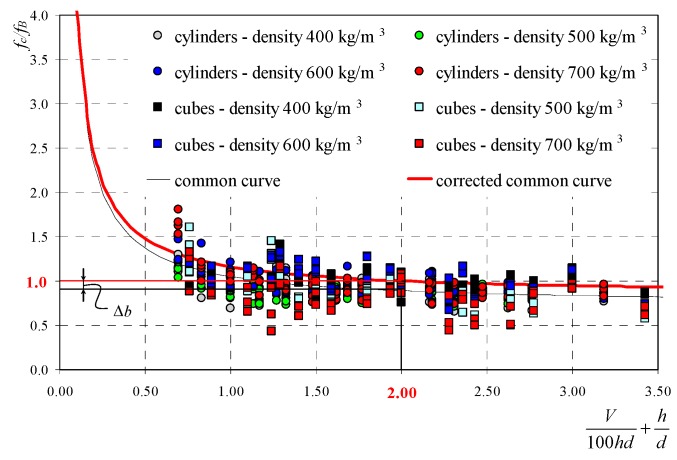
Test results for all core and cube specimens and determined curve of correlation.

The paper [27] compares curve correlations developed for cuboid and cylindrical specimens. Obtained values of curve coefficients *a* and *b* are compared in Table 4. Comparison of test results and the common curve is shown in Figure 4. 

When specimens 100 mm × 100 mm × 100 mm were used, the value of curve dominator was *V*/100*hd* + *h*/*d* = 2, and strength ratios calculated according to equations from Table 4 were *f*_c_/*f*_B_ ≠ 1. To obtain the ratio *f*_c_/*f*_B_ = 1 from normalized specimens, curves needed to be translated in parallel to the intercept axis using the additive correction factor Δ*b* for the common curve:

(13) $$\frac{f_{c}}{f_{B}}=b+\Delta b+\frac{a}{\frac{V}{100hd}+\frac{h}{d}}\to \Delta b=1-b-\frac{a}{2}.$$

To demonstrate the correct scaling of curves, the approximate variance correlation test was applied. This approach is also adequate for linear and non-linear correlations [31]. Statistical values were calculated for each curve from Table 4 using the following formula: $F_{\exp}=\frac{R^{2}}{\left(1-R^{2}\right)}\cdot \frac{f_{2}}{f_{1}}$ where degrees of freedom were *f*_2_ = *n* − *k* − 1 and *f*_1_ = *k* (*k* = 1), and the assumed statistical significance *α* = 5%. The obtained statistical values were compared to critical values from the Fisher–Snedecor tables (*F*_α,f1,f2_). Statistical results are presented in Table 4. Analyses demonstrated that correlations were significant at the assumed statistical significance equal to 5%, thus the proposed model based on the general Neville relation was statistically significant. Besides, descriptive statistics based on the Guillford scale [32] was applied. It describes the correlation degree of individual curves. For concrete with the lowest density, obtained values *R* were sufficient for evaluating the relationship as poor, and for other classes of density *R* > 0.5, correlations could be regarded as moderate and the value of correlation factor as real. For the common curve, the obtained coefficient was *R* = 0.512. Thus, the relationship was moderate and real.

### 2.3. Calibrating a Curve in Air-Dry Conditions

**Figure 5:**
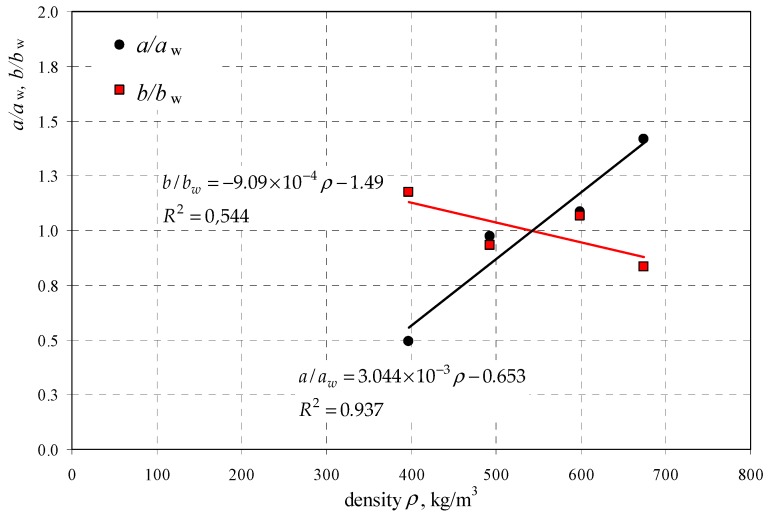
Relative coefficients of curves.

Many curves developed for specific density of AAC were replaced with a curve that was more favourable for diagnostic purposes and could be used to determine the strength of AAC with any density and moisture content. Coefficients *a* and *b* determined for concrete with specific density within the defined ranges and presented in Table 4, as well as coefficients *a*_w_ and *b*_w_ of the common curve were used to develop correlations illustrated in Figure 5.

The following relationships describing curve coefficients as a function of AAC densities were developed on the basis of results shown in Figure 5, using the method of least squares:

(14) $$a=a_{w}\times \left(3.044\times 10^{-3}\rho -0.653\right)=0.321\times \left(3.044\times 10^{-3}\rho -0.653\right),$$

(15) $$b=b_{w}\times \left(9.09\times 10^{-4}\rho -1.49\right)=-0.730\times \left(9.09\times 10^{-4}\rho -1.49\right).$$

where: *a*_w_ = 0.321, and *b*_w_ = 0.730 (see Table 4).

The formation of AAC curve with any density, when *a* and *b* values have been determined, requires a correction for the coefficient *b* which results in the strength ratio obtained from the curve (13) at *V*/(100*hd*) + *h*/*d* = 2.

### 2.4. Calibrating an Empirical Curve in Moisture Conditions

Properties of AAC and ordinary concrete depend on moisture contents [25,33,34], which cause a clear reduction in compressive and tensile strengths, and degradation of insulating parameters. Thus, other tests also focused on the effect of moisture content in AAC, which was a ratio of absorbed water to the mass of dry material:

(16) $$w=\frac{m_{w}-m_{s}}{m_{s}}\cdot 100\%,$$

where *m*_w_ is the mass of wet specimen, and *m*_s_ is the mass of specimen dried until constant weight.

The maximum moisture content (absorbality) *w*_max_ in AAC corresponded to the level of water, at which no further increase in mass *m*_w_ was observed as the effect of passage of (capillary) water. Relative moisture was calculated as the ratio of current and maximum moisture *w*/*w*_max_.

The total number of 127 specimens 100 m × 100 m × 100 m, divided into five six-element series, was prepared from AAC blocks with varying densities. Each specimen was put into containers filled with water to saturate it with water as the effect of passage of (capillary) water. Specimens were weighed every 6 h and moisture content *w* was calculated each time. Maximum moisture content in each type of AAC was assumed to be determined at first, and then specimens were dried until the required moisture content. Strength tests were expected to be performed at the following levels of relative moisture: *w*/*w*_max_ = 100%, 67%, 33%, 23%, 10%, and 0%. Average test results for individual series of specimens are shown in Table 5.

Maximum moisture content in AAC depended on nominal density. At the density increase in the range from *ρ* = 397 kg/m^3^ to 674 kg/m^3^, the maximum moisture content was varying within *w*_max_ = 53.3–89.9%, which made it possible to determine a straight line of the least square in the following form:

(17) $$w_{\max}=-1.23\frac{\rho }{1000}+1.34\;\mathrm{when}\;397{\;\mathrm{kg}/m}^{3}\leq \rho \leq 674{\;\mathrm{kg}/m}^{3}.$$

**Figure 6:**
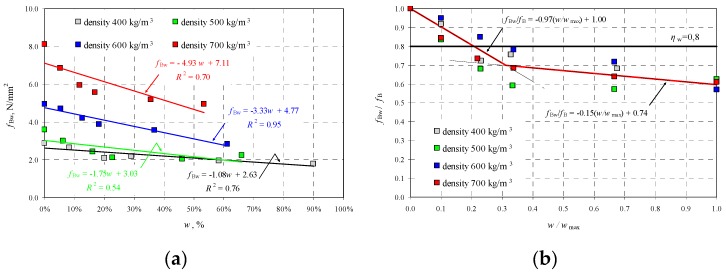
Test results for AAC strength, taking into account moisture level: (**a**) strength *f*_Bw_ as a function of moisture *w*, and (**b**) relative strength of AAC *f*_Bw_/*f*_B_ as a function *w*/*w*_max_.

At each moisture level, destructive tests were performed to determine the strength of wet concrete *f*_Bw_, and the results are illustrated in Figure 6a as a function of moisture *w*. Figure 6b presents the obtained strength values with respect to the strength *f*_B_ of dry (*w* = 0) AAC as a function of relative moisture *w*/*w*_max_.

Two empirical lines were drawn on the basis of obtained results and used to determine the relative strength of AAC as a function of relative moisture in the following form:

(18) $$\frac{f_{\mathrm{Bw}}}{f_{B}}=-0.96\frac{w}{w_{\max}}+1\to f_{\mathrm{Bw}}=f_{B}\left(-0.97\frac{w}{w_{\max}}+1\right)\;\mathrm{when}\;0\leq \frac{w}{w_{\max}}\leq 0.31.$$

(19) $$\frac{f_{\mathrm{Bw}}}{f_{B}}=-0.15\frac{w}{w_{\max}}+0.74\to f_{\mathrm{Bw}}=f_{B}\left(-0.15\frac{w}{w_{\max}}+0.74\right)\;\mathrm{when}\;0.31<\frac{w}{w_{\max}}\leq 1.0.$$

Strength *f*_Bw_ calculated from Equations (18) and (19) included the moisture effect, so it did not require conversion to average normalized compressive strength *f*_b_. 

Figure 6b also shows the value of factor *η*_w_ = 0.8 recommended by the standard EN 772-1 [19], and used to take into consideration the effect of moisture level. The standard recommendation provides the safe reduction of compressive strength only for the moisture level *w*/*w*_max_ = 0.2. Tests on walls with higher moisture content showed that compressive strength could be even reduced by 40%, that is, over twice more than the provisions recommend.

Test results for wet AAC were not thoroughly analyzed with reference to microstructure. It can be assumed that AAC structure will expand the most at moisture content in a range of 30%, and consequently compressive strength will be reduced. To sum it up, determination of compressive strength of the wall *f*_k_ required at first, taking into account varying shape and moisture, in-situ estimation of moisture content, specimen drilling, estimation of density, and compressive strength, and then the conversion relevant to moisture. Compressive strength calculated from the Equations (18) or (19) could be substituted to the Equation (1).

## 3. Ultrasonic Non-Destructive Method

The application of traditional cylindrical transducers may be difficult, as it requires the agent coupling with the tested surface. Tests on very porous and coarse materials, such as AAC, with cylindrical transducers may be also problematic. Measuring the distance of the wave is also difficult, especially if tests are performed only at one side [35,36,37]. Measurements are simpler and easier to perform when transducers having local contact with concrete are applied. Waveguides for this type of transducers are in cone shape or can be formed according to the exponential curve. As energy produced by ultrasound is lower than in cylindrical transducers with a larger contact surface, the spacing of transducers at one-side access to ordinary concrete with density of ca. 2500 kg/m^3^ should not exceed 25 cm, and at both-side access—15 cm [37].

### 3.1. Testing Technique of Specimens

**Figure 7:**
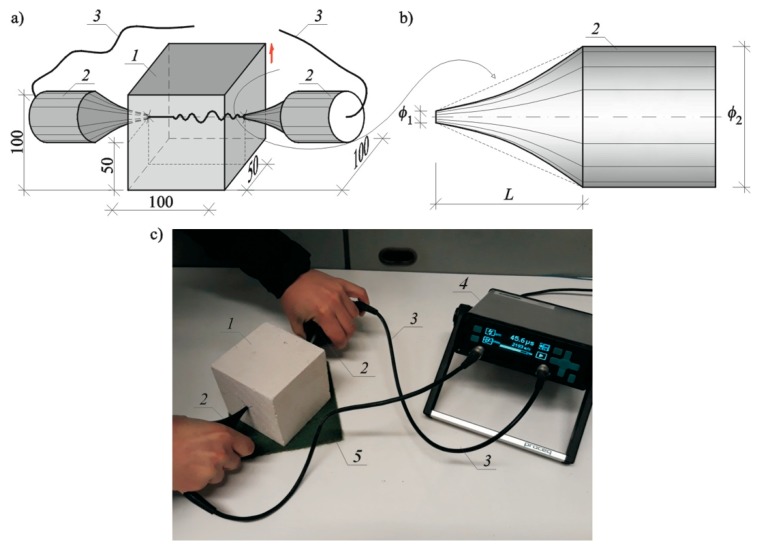
A test stand for measuring ultrasound velocity: (**a**) specimen geometry and elements of the stand (given in millimeters), (**b**) geometry of exponential transducer, and (**c**) a test stand; 1, tested AAC specimen 100 mm × 100 mm × 100 mm; 2, exponential transducers; 3, cables connecting transducers with recording equipment; 4, recording equipment; and 5, an insulating pad.

Non-destructive tests on AAC were performed using the ultrasonic testing, commonly applied for testing strength of concrete [38,39], and testing masonry walls [4,5,6]. Ultrasonic testing was conducted on block specimens 100 mm × 100 mm × 100 mm drilled from masonry units (Figure 7). Wet specimens with relative moisture *w*/*w*_max_ = 100%, 67%, 33%, 23%, and 10%, and specimens dried until constant weight *w*/*w*_max_ = 0% were used in tests. Each series of elements included at least > 20 specimens, and 91 specimens in total were tested.

The PUNDIT LAB instrument (Proceq SA, Schwerzenbach, Switzerland) was utilized for measurements of the ultrasonic pulse velocity. Commercial exponential transducers with the waveguide length *L* = 50 mm, diameters ø_1_ = 4.2 mm and ø_2_ = 50 mm, and frequency 54 kHz were employed. The applied research methodology and equipment was also used for testing also for ultrasonic tomography for concrete [40,41] or masonry [42,43].

Each specimen was put on a pad insulating from shock and outdoor noise, and then transducers were applied to walls and the measurement was made with the transmission method. Transducers were in contact with specimens at an angle of 90° within distance between transducers measured every time with an accuracy up to ±1 mm. Time was measured with an accuracy up to ±0.1 µs. The measurement results are presented in Table 6.

In AAC specimens dried until constant weight, the velocity of ultrasounds was varying from 1847 m/s in concrete of class 400 kg/m^3^ to 2379 m/s in concrete of class 700 kg/m^3^. An increase in P-wave velocity *c*_p_ was also proportional to density increase in wet specimens. 

### 3.2. Calibrating a Curve in Air-Dry Conditions

**Figure 8:**
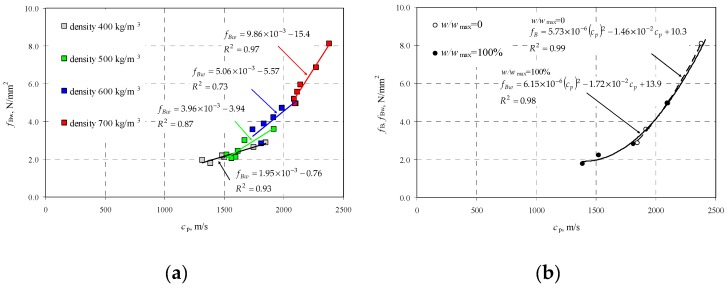
Results from P-waves velocity testing: (**a**) compressive strength of AAC including density classes, and (**b**) AAC strength in wet concrete (*f*_Bw_) and totally dry concrete (*f*_B_).

Performed tests showed that density and relative moisture affected the velocity of P-waves in AAC. By performing steps described in Section 2.2, at first the correlation curve was determined which presented ultrasound velocity in AAC specimens in air-dry conditions as a function of compressive strength *f*_B_. At the beginning, the curve representing the relationship between the average measured ultrasound velocity as a function of compressive strength *f*_Bw_ of wet AAC, grouping results by AAC density (Figure 8a). Higher sound velocity was found in concrete with greater density and compressive strength. Linear dependence, equations of which are illustrated in Figure 8a, are adequately precise approximations. Figure 8b illustrates results for compressive strength and corresponding ultrasound velocity of dry AAC (*w*/*w*_max_ = 0%), selected from each density class of AAC. Then, the relationship *c*_p_–*f*_B_ was calculated with the least square method. For example, Figure 8b also shows the relationship of concrete with maximum moisture content (*w*/*w*_max_ = 100%), obtained similarly.

For concrete with moisture content *w*/*w*_max_ = 0%, the following empirical relationship was obtained:

(20) $$f_{B}=a{\left(c_{p}\right)}^{2}+bc_{p}+c=5.73\times 10^{-6}{\left(c_{p}\right)}^{2}-1.46\times 10^{-2}c_{p}+10.3,\;\mathrm{when}\;1847\;m/s<c_{p}\leq 2379\;m/s.$$

Curve (20) covers results from testing all densities of AAC, where the obtained coefficient of correlation is *R*^2^ = 0.98.

### 3.3. Calibrating a Curve in Moisture Conditions

**Figure 9:**
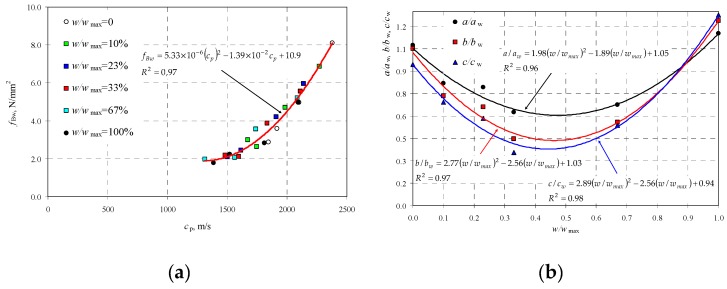
Results from ultrasound velocity testing: (**a**) common curve *f*_Bw_–*c*_p_ for all AAC densities and moisture levels, and (**b**) equations for curve coefficients at varying moisture content in AAC *f*_Bw_.

The practical use of obtained test results required the common curve covering both the varying density of AAC and the moisture impact. For this purpose, the common curve including all moisture levels *w*/*w*_max_ and densities, was found with the least square method (Figure 9a). The equation of the common curve was:

(21) $$f_{\mathrm{Bw}}=a_{w}{\left(c_{p}\right)}^{2}+b_{w}c_{p}+c_{w}\to f_{\mathrm{Bw}}=5.33\cdot 10^{-6}{\left(c_{p}\right)}^{2}-1.39\cdot 10^{-2}c_{p}+10.9\;\mathrm{when}\;1315\;m/s<c_{p}\leq 2379\;m/s.$$

Then, equations for individual curves were developed with reference to AAC density. Test results are presented in Table 7. The obtained coefficient values were compared to coefficients *a*_w_, *b*_w_, and *c*_w_ for the common curve, and then plotted to the graph Figure 9b. 

A parabolic relation of the compressive strength of wet AAC *f*_bw_–*c*_p_ illustrated in Figure 9a was the same as for ordinary concrete [1]. Linear and parabolic relations were obtained for solid brick [21,44]. Generally, the result was similar to predictions. Taking into account that ultrasound velocity depends on the modulus of elasticity E, Poisson’s ratio ν, and density ρ, and connected with the relationship $c_{p}=\sqrt{E\left(1-\nu \right)/\rho \left(1+\nu \right)\left(1-2\nu \right)}$, it was easily demonstrated that greater density caused by moisture content resulted in an increase in the modulus of elasticity. Obtained curves shown in Figure 9b are statistic. High *R*^2^ values represent non-linear correlations. All curves had the minimum at moisture content in the range of *w*/*w*_max_ = 0.4–0.5. As a consequence, a difference in results with reference to the common curve will be the biggest. The curve obtained at this moisture content *f*_bw_–*c*_p_ was likely to be shifted downwards. Further studies require additional tests at moisture content in the range of *w*/*w*_max_ = 0.4–0.5. 

The method of least squares gave the following forms of empirical curves used to determine coefficients of the relationship *f*_Bw_–*c*_p_ for AAC with any moisture level and density:

(22) $$\frac{a}{a_{w}}=1.99{\left(\frac{w}{w_{\max}}\right)}^{2}-1.89\frac{w}{w_{\max}}+1.05,\;R^{2}=0.96,$$

(23) $$\frac{b}{b_{w}}=2.77{\left(\frac{w}{w_{\max}}\right)}^{2}-2.56\frac{w}{w_{\max}}+1.03,\;R^{2}=0.97,$$

(24) $$\frac{c}{c_{w}}=2.89{\left(\frac{w}{w_{\max}}\right)}^{2}-2.56\frac{w}{w_{\max}}+0.94,\;R^{2}=0.98.$$

Calculated coefficients *a*, *b*, and *c* should be put into the equation:

(25) $$f_{\mathrm{Bw}}=a{\left(c_{p}\right)}^{2}+bc_{p}+c,\;\mathrm{when}\;1315\;m/s<c_{p}\leq 2379\;m/s.$$

which gives the general form of the basic curve for AAC. In practice, ultrasonic testing should be associated with destructive tests for graduation. In this case, further steps can follow rules specified in the European standard EN 13791 [1] for ordinary concrete.

## 4. Procedure Algorithm for Determining Characteristic Compressive Strength of Masonry

Proposed empirical procedure for determining characteristic compressive strength of masonry with semi-non-destructive and non-destructive techniques can be described with the following steps shown in Table 8. 

## 5. Conclusions

The preformed tests confirmed the effect of specimen shape on compressive strength in the analyzed type of autoclaved aerated concrete and on the method of specimen failure. Regardless of AAC density, compressive strength determined at specific volumes and slenderness was found to be similar to the strength of standard specimens. The greatest strength was found in specimens with the smallest volume. Compressive strength of specimens with the greatest volume was much lower than in case of standard cube specimens with dimensions of 100 mm × 100 mm × 100 mm. 

Maximum moisture content was increasingly reversely proportional to AAC density, and moisture significantly reduced strength with reference to the strength of AAC tested in air-dry conditions. The greatest 30% reduction in compressive strength was observed at moisture content *w* = 0–30%. This observation was particularly important because moisture content of masonry is ca. 10–15%. Higher moisture levels caused a drop in strength by 10%. AAC moisture coefficient *η*_w_ = 0.8 recommended by the standard PN-EN 772-1 may give dangerously overestimated strength of masonry with moisture content *w* > 20%. 

The non-destructive ultrasonic testing demonstrated the profound effect of density and moisture. An increase in P-waves velocity was proportional to density of AAC (maximum velocity was 2379 m/s in concrete with density of 700 kg/m^3^, minimum velocity was 1847 m/s in concrete with density of 400 kg/m^3^). Increasing density of AAC caused a significant reduction of the velocity. 

Two complementary techniques were used. The first semi-non-destructive test can determine compressive strength of masonry units based on testing specimens of any shape. This technique can be used independently if at least 18 specimens can be prepared (cf. EN 13791:2009 [1])—like for concrete. The second is the non-destructive ultrasound method, which cannot be generally used without scaling the obtained curve. However, the great advantage of this solution is the reduced number of specimens to be prepared and scaled. The number of six drilled cores or cuboid specimens can be assumed as minimum. After scaling the curve, measurements can be made at any number of points and AAC strength can be determined. Performed tests indicated the impact of AAC density and moisture on both compressive strength and ultrasound velocity. These methods have some material limitations with reference to density $397{\;\mathrm{kg}/m}^{3}\leq \rho \leq 674{\;\mathrm{kg}/m}^{3}$ and ultrasound velocity $1847\;m/s<c_{p}\leq 2379\;m/s$.

## Figures and Tables

**Table 1:** Results from compressive tests performed on core (cylindrical) specimens.

No.	Class of Density kg/m^3^	Specimen Type	Dimensions, mm		No. of Specimens *n*	Average Compressive Strength *f*_ci_, N/mm^2^	Standard Deviation $s=\sqrt{\frac{{\left(f_{i}-f_{\mathrm{ci}}\right)}^{2}}{n-1}},\;N/{\mathrm{mm}}^{2}$	C.O.V $\frac{s}{f_{\mathrm{ci}}},$ %
			Diameter, *ø*	Height, *h*				
1	2	3	4	5	6	7	8	9
1	400	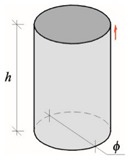	150	150	6	2.84	0.40	14
2				301	3	2.33	0.13	6
3				76	3	2.70	0.06	2
4			97.6	97.8	4	2.61	0.29	11
5				195	3	2.16	0.21	10
6				49	4	2.81	0.39	14
7			79.4	79.2	3	2.53	0.25	10
8				159	3	2.26	0.26	12
9				40.6	3	2.85	0.17	6
10			61	61	4	2.77	0.17	6
11				121.8	3	2.65	0.12	5
12				31.8	5	2.51	0.39	15
13			39.5	40	5	2.82	0.42	15
14				79	4	2.28	0.34	15
15				20.5	4	2.60	0.35	14
16			25	24.4	3	2.33	0.30	13
17				49.2	3	2.69	0.42	16
18				12.5	3	3.56	0.33	9
1	500		150	150	6	2.94	0.25	9
2				301	3	3.28	0.18	6
3				76	3	3.01	0.12	4
4			97.6	97.8	4	2.88	0.16	6
5				195	3	3.09	0.06	2
6				49	4	3.15	0.29	9
7			79.4	79.2	3	3.30	0.12	4
8				159	3	2.90	0.21	7
9				40.6	4	3.27	0.45	14
10			61	61	4	3.21	0.23	7
11				121.8	3	3.17	0.26	8
12				31.8	5	3.19	0.18	6
13			39.5	40	4	2.94	0.30	10
14				79	4	2.89	0.32	11
15				20.5	4	3.63	0.36	10
16			25	24.4	4	2.91	0.27	9
17				49.2	3	3.16	0.18	6
18				12.5	4	4.06	0.25	6
1	600	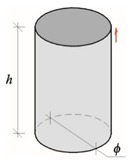	150	150	5	5.06	0.36	7
2				301	3	4.23	0.21	5
3				76	3	5.11	0.68	13
4			97.6	97.8	4	4.49	0.22	5
5				195	3	4.26	0.18	4
6				49	4	5.01	0.61	12
7			79.4	79.2	4	4.43	0.09	2
8				159	3	4.73	0.25	5
9				40.6	4	5.14	0.52	10
10			61	61	4	4.65	0.47	10
11				121.8	3	4.54	0.16	3
12				31.8	5	5.19	0.66	13
13			39.5	40	4	4.87	0.53	11
14				79	3	4.18	0.31	8
15				20.5	4	6.00	0.81	14
16			25	24.4	4	5.17	0.27	5
17				49.2	3	4.79	0.64	13
18				12.5	4	6.88	0.76	11
1	700		150	150	5	7.12	0.96	14
2				301	3	7.25	0.56	8
3				76	4	7.69	0.63	8
4			97.6	97.8	4	7.37	0.76	10
5				195	3	7.22	0.42	6
6				49	4	7.93	0.28	4
7			79.4	79.2	3	6.77	0.35	5
8				159	3	7.25	0.57	8
9				40.6	4	8.87	0.36	4
10			61	61	4	7.25	1.04	14
11				121.8	3	7.05	0.51	7
12				31.8	5	8.57	0.35	4
13			39.5	40	3	7.55	0.32	4
14				79	3	7.21	1.08	15
15				20.5	4	9.18	0.77	8
16			25	24.4	3	7.66	0.77	10
17				49.2	3	7.73	0.40	5
18				12.5	4	13.42	0.95	7

**Table 2:** Results from compressive tests performed on cuboid specimens.

No.	Class of Density kg/m^3^	Specimen Type	Dimensions, mm			No. of Specimens *n*	Average Compressive Strength *f*_ci_, N/mm^2^	Standard Deviation $s=\sqrt{\frac{{\left(f_{i}-f_{\mathrm{ci}}\right)}^{2}}{n-1}},$ N/mm^2^	C.O.V $\frac{s}{f_{\mathrm{ci}}},$ %
			Width, *d*	Thickness, *b*	Height, *h*				
1	2	3	4	5	6	7	8	9	10
1	400	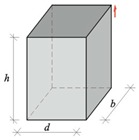	143	143	143	3	2.80	0.18	6
2					72	3	2.91	0.14	5
3					285	3	2.47	0.05	2
4			100	100	100 *	6	2.88	0.36	12
5					50	3	2.59	0.24	9
6					200	3	3.16	0.13	4
7			80	80	80	3	3.12	0.23	7
8					39	3	3.60	0.44	12
9					158	3	2.71	0.15	5
10			59	59	59	3	2.99	0.11	4
11					30	3	3.16	0.17	5
12					121	3	2.98	0.08	3
13			40	40	40	3	2.85	0.07	3
14					19.6	3	3.02	0.07	2
15					78.5	3	2.77	0.41	15
16			24	24	24	3	2.80	0.20	7
17					12.5	3	3.23	0.56	17
18					49	3	2.43	0.26	11
1	500		143	143	143	3	2.33	0.28	12
2					72	3	3.74	0.06	2
3					285	3	2.16	0.11	5
4			100	100	100 *	6	3.59	0.13	4
5					50	3	3.29	0.13	4
6					200	3	3.40	0.06	2
7			80	80	80	3	3.31	0.15	5
8					39	3	3.67	0.11	3
9					158	3	2.48	0.22	9
10			59	59	59	3	2.83	0.09	3
11					30	3	3.20	0.55	17
12					121	3	2.94	0.17	6
13			40	40	40	3	2.90	0.04	1
14					19.6	3	3.28	0.21	6
15					78.5	3	2.77	0.44	16
16			24	24	24	3	4.78	0.39	8
17					12.5	3	4.92	0.90	18
18					49	3	1.79	0.10	6
1	600	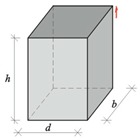	143	143	143	3	3.97	0.10	2
2					72	3	5.69	0.10	2
3					285	3	3.58	0.25	7
4			100	100	100 *	6	4.95	0.35	7
5					50	3	5.80	0.35	6
6					200	3	5.34	0.61	11
7			80	80	80	3	6.01	0.75	12%
8					39	3	6.60	0.12	2
9					158	3	4.45	0.19	4
10			59	59	59	3	4.58	0.08	2
11					30	3	5.85	0.04	1
12					121	3	4.84	0.09	2
13			40	40	40	3	5.81	0.41	7
14					19.6	3	5.06	0.17	3
15					78.5	3	5.65	0.20	4
16			24	24	24	3	6.02	0.74	12
17					12.5	3	6.30	0.19	3
18					49	3	4.19	0.91	22
1	700		143	143	143	3	4.88	1.03	21
2					72	3	7.21	0.27	4
3					285	3	5.28	0.44	8
4			100	100	100 *	6	8.11	0.58	7
5					50	3	7.02	1.07	15
6					200	3	7.56	0.25	3
7			80	80	80	3	6.31	0.27	4
8					39	3	8.79	0.89	10
9					158	3	6.50	0.98	15
10			59	59	59	3	5.76	0.34	6
11					30	3	6.31	1.10	17
12					121	3	4.65	0.95	20
13			40	40	40	3	5.48	0.52	10
14					19.6	3	7.00	0.22	3
15					78.5	3	6.71	0.29	4
16			24	24	24	3	5.38	1.98	37
17					12.5	3	9.37	1.96	21
18					49	3	4.89	1.66	34

* cube specimens according to PN-EN 771-4:2012 [28] used to determine compressive strength *f*_B_.

**Table 3:** Test results for AAC density.

No.	Nominal Class of Density, kg/m^3^	No. of Cuboid Specimens (see Table 2)	Average Density, kg/m^3^	Standard Deviation *s*, kg/m^3^	C.O.V., %
1	400	57	397	22.01	6
2	500	57	492	15.86	3
3	600	57	599	13.39	2
4	700	57	674	19.83	3

**Table 4:** Comparison of coefficients and equations of empirical curves.

Density Range of AAC, Average Density *ρ*, (Nominal Class of Density) kg/m^3^	Coefficient for Curve		*R*	Additive Correction Factor Δ*b*	Corrected Coefficient for Curve *b*_kor_	Curve Equation	*n*	$\begin{array}{c} F_{\exp}\\ F_{\alpha ,f_{1},f_{2}} \end{array}$
	*a*	*b*						
from 375 to 446 397, (400)	0.159	0.857	0.324	0.06	0.921	$\frac{f_{c}}{f_{B}}=0.921+\frac{0.159}{\frac{V}{100hd}+\frac{h}{d}}$	123	$\begin{array}{c} 14.19\\ 3.919 \end{array}$
from 462 to 532 492, (500)	0.312	0.682	0.533	0.16	0.844	$\frac{f_{c}}{f_{B}}=0.844+\frac{0.312}{\frac{V}{100hd}+\frac{h}{d}}$	125	$\begin{array}{c} 48.81\\ 3.918 \end{array}$
from 562 to 619 599, (600)	0.349	0.779	0.612	0.05	0.826	$\frac{f_{c}}{f_{B}}=0.826+\frac{0.349}{\frac{V}{100hd}+\frac{h}{d}}$	124	$\begin{array}{c} 73.06\\ 3.919 \end{array}$
from 655 to 725 674, (700)	0.454	0.608	0.614	0.16	0.773	$\frac{f_{c}}{f_{B}}=0.773+\frac{0.454}{\frac{V}{100hd}+\frac{h}{d}}$	122	$\begin{array}{c} 72.62\\ 3.920 \end{array}$
common curve	*a*_w_ = 0.321	*b*_w_ = 0.730	0.512	0.11	0.840	$\frac{f_{c}}{f_{B}}=0.840+\frac{0.321}{\frac{V}{100hd}+\frac{h}{d}}$	494	$\begin{array}{c} 174.8\\ 3.860 \end{array}$

**Table 5:** Test results for AAC with varying moisture content.

No.	Density Range of AAC, Average Density *ρ*, (nominal class of density) kg/m^3^	Average Moisture Content *w*, %	Average Relative Moisture *w*/*w*_max_	Average Compressive Strength *f*_Bw_, N/mm^2^	Standard Deviation, *s*, N/mm^2^	COV, %	Average Relative Compressive Strength *f*_Bw_/*f*_B_
1	from 375 to 446 397, (400)	0	0	2.88 *	0.36	12	1.0
2		8.3	0.10	2.64	0.21	8	0.92
3		20.1	0.23	2.09	0.11	5	0.72
4		29.1	0.33	2.18	0.16	8	0.76
5		58.3	0.67	1.96	0.14	7	0.68
6		89.9	1.00	1.78	0.13	7	0.62
7	from 462 to 532 492, (500)	0	0	3.59 *	0.13	4	1.0
8		6.2	0.10	3.00	0.22	7	0.84
9		16.2	0.23	2.44	0.49	20	0.68
10		22.8	0.33	2.12	0.21	10	0.59
11		46.1	0.67	2.06	0.29	14	0.57
12		66.0	1.00	2.24	0.23	10	0.62
13	from 562 to 619 599, (600)	0	0	4.95 *	0.35	7	1.0
14		5.40	0.10	4.71	0.49	10	0.95
15		12.6	0.23	4.21	0.38	9	0.85
16		18.2	0.34	3.88	0.52	13	0.78
17		58.3	0.67	1.96	0.33	9	0.68
18		61.1	1.00	2.82	0.28	10	0.57
19	from 655 to 725 674, (700)	0	0	8.11 *	0.58	7	1.0
20		5.30	0.10	6.86	0.63	9	0.85
21		11.7	0.22	5.96	0.71	12	0.74
22		16.8	0.34	5.56	0.58	10	0.69
23		46.1	0.67	2.06	0.70	13	0.57
24		53.3	1.00	4.95	0.41	8	0.61

* *f*_B_—compressive strength of dry AAC, when *w* = 0.

**Table 6:** Test results for ultrasound velocity in AAC with varying moisture content.

No.	Density Range of AAC, Average Density *ρ*, (nominal class of density) kg/m^3^	*w*/*w*_max_	Average Path Length *L*, mm	Average Passing Time of Wave *t*, µs	Average P-Wave Velocity *c*_p_ = *L*/*t*, m/s	*N*	Standard Deviation, $s=\sqrt{\frac{{\left(c_{\mathrm{pi}}-c_{p}\right)}^{2}}{n-1}}\;s,\;m/s$	C.O.V., $\frac{s}{f_{\mathrm{ci}}}\;\%$
1	from 375 to 446 397, (400),	0	100.2	54.3	1847	21	35.9	1.9
2		0.10		57.4	1746	21	24.0	1.4
3		0.23		67.0	1501	21	37.7	2.5
4		0.33		67.6	1483	21	32.8	2.2
5		0.67		76.5	1315	21	25.6	1.9
6		1.00		72.7	1384	21	44.5	3.2
7	from 462 to 532 492, (500),	0	100.4	52.4	1917	23	51.4	2.7
8		0.10		56.3	1671	23	28.3	1.7
9		0.23		62.3	1614	23	33.6	2.1
10		0.33		63.0	1595	23	34.7	2.2
11		0.67		64.4	1562	23	70.2	4.5
12		1.00		62.0	1520	23	43.9	2.9
13	from 562 to 619 599, (600),	0	100.2	47.7	2101	24	49.7	2.4
14		0.10		50.5	1985	24	41.7	2.1
15		0.23		52.5	1910	24	59.6	3.1
16		0.34		54.7	1832	24	52.7	2.9
17		0.67		58.0	1738	24	69.1	4.0
18		1.00		55.6	1812	24	58.3	3.2
19	from 655 to 725 674, (700),	0	100.5	42.2	2379	23	46.2	1.9
20		0.10		44.3	2269	23	43.1	1.9
21		0.22		47.0	2139	23	52.4	2.4
22		0.34		47.6	2111	23	51.5	2.4
23		0.67		48.4	2085	23	56.1	2.7
24		1.00		48.2	2094	23	28.3	1.4

**Table 7:** Comparison of coefficients and equations of empirical curves.

*w*/*w*_max_	Curve Coefficient			*R* ^2^	Curve Equation
	*a*	*b*	*c*		
0	5.73 × 10^−6^	−1.46 × 10^−2^	10.30	0.99	$f_{\mathrm{Bw}}=5.73\times 10^{-6}{\left(c_{p}\right)}^{2}-1.46\times 10^{-2}c_{p}+10.3$
0.1	4.37 × 10^−6^	−1.02 × 10^−2^	7.56	0.97	$f_{\mathrm{Bw}}=4.37\times 10^{-6}{\left(c_{p}\right)}^{2}-1.02\times 10^{-2}c_{p}+7.56$
0.23	4.22 × 10^−6^	−9.19 × 10^−3^	6.35	0.99	$f_{\mathrm{Bw}}=4.22\times 10^{-6}{\left(c_{p}\right)}^{2}-9.19\times 10^{-3}c_{p}+6.35$
0.33	3.33 × 10^−6^	−6.21 × 10^−3^	3.88	0.98	$f_{\mathrm{Bw}}=3.33\times 10^{-6}{\left(c_{p}\right)}^{2}-6.21\times 10^{-3}c_{p}+3.88$
0.67	3.59 × 10^−6^	−7.75 × 10^−3^	5.84	0.95	$f_{\mathrm{Bw}}=3.59\times 10^{-6}{\left(c_{p}\right)}^{2}-7.75\times 10^{-3}c_{p}+5.84$
1	6.15 × 10^−6^	−1.72 × 10^−2^	13.90	0.98	$f_{\mathrm{Bw}}=6.15\times 10^{-6}{\left(c_{p}\right)}^{2}-1.72\times 10^{-3}c_{p}+13.90$
common curve	*a*_w_ = 5.33 × 10^−6^	*b*_w_ = −1.39 × 10^−2^	*c*_w_ = 10.90	0.97	$f_{\mathrm{Bw}}=5.33\times 10^{-6}{\left(c_{p}\right)}^{2}-1.39\times 10^{-2}c_{p}+10.9$

**Table 8:** Procedure algorithm for determining characteristic compressive strength of masonry with semi-NDT and NDT techniques.

Step	Description			
	Semi-Non-Destructive Testing	Reference	Non-Destructive (ultrasonic) Testing	Reference
1	Determining moisture content by weight *w* in AAC at the tested (in-situ) point	Equation (16)	Determining moisture content by weight *w* in AAC at the tested point	Equation (16)
2	Calculating maximum moisture content *w*_max_ in AAC	Equation (17)	Calculating maximum moisture content *w*_max_ in AAC	Equation (17)
3	Drilling specimens from AAC, drying them until constant weight and calculating density *ρ*	-	Determining P-waves velocity (*c*_p_ = *L*/*t*,) using the transmission method after measuring the path length *L* and time *t*.	-
4	Calculating coefficients *a* and *b* of the empirical curve	Equations (14) and (15)	Calculating coefficients *a* and *b* of the empirical curve	Equations (22)–(24)
5	Calculating the correction factor Δ*b*	Equation (13)	Calculating compressive strength of AAC *f*_Bw_ acc. to the curve	Equation (25)
6	Performing destructive tests and determining compressive strength of dry AAC *f*_c_	-	Graduating the curve according to the standard EN 13791:2008	-
7	Calculating compressive strength *f*_B_ acc. to the corrected curve	Equation (13)	Calculating compressive strength of AAC *f*_Bw_ acc. to the graduated curve	-
8	Calculating compressive strength *f*_Bw_ depending on moisture content in AAC	Equations (18) and (19)	Calculating characteristic compressive strength of AAC masonry	Equation (1)
9	Calculating characteristic compressive strength *f*_k_ of AAC masonry	Equation (1)		
